# What cultural values determine student self-efficacy? An empirical study for 42 countries and economies

**DOI:** 10.3389/fpsyg.2023.1177415

**Published:** 2023-06-20

**Authors:** Rui Jin, Rongxiu Wu, Yuyan Xia, Mingren Zhao

**Affiliations:** ^1^Faculty of Education, Shenzhen University, Shenzhen, Guangdong, China; ^2^Science Education Department Harvard-Smithsonian Center for Astrophysics, Harvard University, Cambridge, MA, United States; ^3^Department of Education Policy and Evaluation, University of Kentucky, Lexington, KY, United States

**Keywords:** cultural values, student self-efficacy, PISA 2018, alignment method, classification and regression tree

## Abstract

Self-efficacy is a vital personal characteristic for student success. However, the challenge of cross-cultural comparisons remains as scalar invariance is hard to be satisfied. Also, it is unclear how to contextually understand student self-efficacy in light of cultural values in different countries. This study implements a novel alignment optimization method to rank the latent means of student self-efficacy of 308,849 students in 11,574 schools across 42 countries and economies that participated in the 2018 Program in International Student Assessment. We then used classification and regression trees to classified countries with differential latent means of student self-efficacy into groups according to Hofstede’s six cultural dimensions theory. The results of the alignment method recovered that Albania, Colombia, and Peru had students with the highest mean self-efficacy, while Slovak Republic, Moscow Region (RUS), and Lebanon had the lowest. Moreover, the CART analysis indicated a low student self-efficacy for countries presenting three features: (1) extremely high power distance; (2) restraint; and (3) collectivism. These findings theoretically highlighted the significance of cultural values in shaping student self-efficacy across countries and practically provided concrete suggestions to educators on which countries to emulate such that student self-efficacy could be promoted and informed educators in secondary education institutes on the international expansion of academic exchanges.

## 1. Introduction

Student self-efficacy has been defined as the belief that students believe they have the ability to engage in learning activities and deal with tasks, especially in an adverse situation (Bandura, 1977; Waddington, 2023). Previous studies have documented that self-efficacy impacts multiple student academic performance, such as math score or math problem-solving ability (Klassen and Klassen, 2018; Uchida et al., 2018; Wang et al., 2022; Yang et al., 2022), reading score (Graham et al., 2019; Liu et al., 2020; Zhou et al., 2020), and English learning for foreigners (Wu et al., 2013; Wang and Sun, 2020; Xu et al., 2022). Moreover, a high level of self-efficacy benefits emotional and cognitive outcomes. Extensive literature has documented that self-efficacy positively stirs students’ intrinsic motivations in almost all aspects of learning and related tasks and activities (Chung et al., 2021; Ma, 2021; Tannert and Gröschner, 2021; Wang et al., 2022).

Recognizing the importance of self-efficacy, large-scale international assessments, such as the Program for International Student Assessment (PISA), have measured it in their investigations (OECD, 2019a,b). PISA 2018 developed a measurement tool containing five specific items to assess student perceptions of self-efficacy (Schleicher, 2019). Using the collected data from the students across 79 participating countries, the PISA team made a comparison to see which countries had high/low ratings for each self-efficacy item by calculating the percentage of students who strongly agreed or agreed with each item. However, before making valid comparisons across countries, it is imperative to ensure that the scale works in the same manner across all the participating countries (Whisman and Judd, 2016; Xu and Tracey, 2017).

As a meaningful cross-country comparison requires scalar invariance that is difficult to establish (Long and Brekke, 1999; Davidov, 2009), researchers have recently been gradually applying the alignment approach first introduced by Asparouhov and Muthén (2014) to evaluate measurement invariance across multiple groups. A prominent advantage of the alignment approach is that it merely requires configural invariance, which could significantly lower the barrier of cross-country comparison. Thus, the first goal of the current research is to assess whether the configural invariance of the self-efficacy scale across the countries holds, using data from PISA 2018. Further, we compare the self-efficacy factor mean scores by employing the novel alignment optimization method.

Understanding the factors that determine student self-efficacy is an important topic in the education field. These constitute a set of student, family, teacher, and school-level factors, such as student cognitive activation (Li et al., 2021), family socioeconomic status (Ma, 2021), teaching approaches (Gao et al., 2020), and school discipline and safety (Chen et al., 2021). With the increasingly globalized world in which students have more opportunities to engage in culturally diverse programs, how student self-efficacy might be influenced by their cultural background has been receiving more attention (Khine and Nielsen, 2022, p. 112; Oettingen and Zosuls, 2006). Based on Hofstede’s six cultural dimensions theory (2005), previous studies have documented the mediating effects of cultural values (e.g., individualism) on the associations between student self-efficacy and several independent variables (e.g., teacher self-efficacy) (Bonneville-Roussy et al., 2019), but few studies have comprehensively assessed six types of cultural values and which is the most influential. Elucidating the complicated mechanism of cultural differences in shaping student self-efficacy might produce country-specific information on how student self-efficacy operates (Bonneville-Roussy et al., 2019; Wan et al., 2022). Therefore, using countries’ ranking by student self-efficacy, we use classification and regression tree analysis to classify 42 countries into small groups that share similar Hofstede’s six cultural dimensions, and deconstruct the intricate relationships among these cultural values.

## 2. Literature review

### 2.1. Measurement bias and invariance in large-scale comparisons

Whenever one construct is compared in a large-scale assessment, there is always a risk of measurement bias being introduced, reducing the validity of scientific conclusions (Van de Vijver and Leung, 2011). Three types of biases have been widely discussed (Van der Vijver and Rothmann, 2004): construct bias, method bias, and item bias. Construct bias occurs when a concept measures groups differently (McDermott et al., 2020; Gerstein, 2021). For example, a question asks patients to illustrate the characteristics of a good nurse. These characteristics are quite variable across cultures, and an evaluator from a particular culture might have a different perspective on nurses from cultural groups different than their own. Method bias arises from using inappropriate investigation techniques across groups (Kock et al., 2021). For instance, an example of administration bias (one type of method bias) is that miscommunication is almost certain to occur between testers and testees from different cultural backgrounds (van de Vijver, 2002). Item bias refers to differential item functioning, and occurs when different groups respond differently to particular items (Ellis, 1989; Ross et al., 2023).

The elimination of measurement bias is a precondition for achieving cross-cultural measurement invariance; hence, these two constructs (i.e., measurement bias versus measurement invariance) may be seen as opposite sides of the same coin (He and van de Vijver, 2012). Scholars have proposed three types of measurement invariance (Meredith, 1993; Steenkamp and Baumgartner, 1998): configural, metric, and scalar. Configural invariance means the same overall factor of self-efficacy holds for all countries (Yue et al., 2022). It suggests that respondents from different groups adopt the same theoretical framework to respond to a set of items in a scale (Cheung and Rensvold, 2002). Metric invariance requires that the factor loadings are identical across all countries, which indicates that items share equivalent meaning in terms of their relationship to the factor, across groups (Milfont and Fischer, 2010; Jovanović et al., 2022). Scalar invariance is the most constrained, with both loadings and intercepts being identical across all countries (Meredith, 1993; Arrindell et al., 2022). Following the configural invariance level, each level of measurement invariance requires evidence supporting invariance at the prior level (Millsap, 2012). Generally speaking, a valid cross-cultural comparison requires scalar invariance that is often rejected (Asparouhov and Muthén, 2014). Therefore, it is critical to develop an approach to lower the barrier.

### 2.2. Alignment approach

As scalar invariance is rarely achieved in large cross-national comparisons (Davidov et al., 2014; Hoth et al., 2022), researchers have introduced a method called partial measurement invariance (Byrne, 1989; Byrne and van de Vijver, 2017). That is, through a systematic examination, scholars identify the items with the most invariance and fix their parameters across groups, while allowing other items to be freely estimated. However, when the number of items increases, this method may be error-prone as it conducts an exploratory process (Vandenberg and Lance, 2000). Multi-pairwise mean comparisons method has further been proposed (Zieger et al., 2019). This method fits a factor analysis model on the data from one group and then compares the determined latent mean with each latent mean of other comparable groups one by one, during which three types of measurement invariance were considered. However, as the number of groups increases, both partial measurement invariance and multi-pairwise mean comparisons could become very labor-intensive and might not identify the group with the highest/lowest latent mean (Zakariya et al., 2020; Wu et al., 2022).

In 2014, Asparouhov and Muthén (2014) developed a new approach for multi-group confirmatory factor analysis (CFA), called the alignment method. Its most significant strength is that it only requires configural invariance rather than exact measurement invariance (i.e., scalar invariance), which significantly lowers the barrier of the group comparisons (Asparouhov and Muthén, 2014). By automatically testing measurement invariance through multi-groups with expected non-invariance, the alignment method is not only able to estimate factor means, factor loadings, and item parameters across groups (Wu et al., 2022). but also tests their invariance to identify the most invariant and non-invariant items (Asparouhov and Muthén, 2014).

Alignment method has been applied effectively in testing measurement invariance across groups (e.g., Munck et al., 2018; Ding et al., 2022), creating measurement models (e.g., Tay et al., 2017; Glassow et al., 2021), and making cross-countries comparisons (e.g., Zakariya et al., 2020; Zakariya, 2021; Wu et al., 2022). However, after an exhaustive search of previous studies, we did not find its application in cross-country comparisons of student self-efficacy, which is a research gap this study attempts to fill.

### 2.3. Addressing self-efficacy from a cultural perspective

The global population of students who attend study-abroad programs has increased enormously (Isabelli-García et al., 2018). The extant literature suggests that even a short-term summer stay abroad (3–4 weeks) during secondary education could be beneficial for students’ development in language learning and intercultural competence (Llanes and Muñoz, 2009; Isabelli-García et al., 2018). However, compared to domestic students of host countries, international students encounter more challenges from the language barrier and different cultural values.

Cultural values refer to “trans-situational goals… that serve as guiding principles in the life of a person or other social entity” (Schwartz, 1994, p. 21). Hofstede et al. (2005) proposed a seminal theoretical framework to elaborate cultural values across six dimensions: (1) power distance, measuring the degree of inequality in a society. In societies with a large power distance, inequalities are expected and desired; (2) individualism versus collectivism, reflecting the degree of loose ties between individuals. Students in collectivistic countries generally emerge with a lower level of self-efficacy than those in individualistic countries; (3) masculinity versus femineity. Feminine culture values relationships and quality of life, but masculine culture values challenge, competition, and advancement; (4) uncertainty avoidance, measuring the anxiety level of a country. A country with strong uncertainty avoidance often reports high stress and people feel less happy; (5) long-term versus short-term orientation, the former meaning the cultivation of values that are directed toward future benefits, particularly persistence and thriftiness, while the latter emphasizes the cultivation of qualities connected to the past and the present, particularly a reverence for tradition and fulfillment of social duties; and (6) indulgence versus restraint, measuring personal impulse and desire control. High levels of indulgence suggest that a culture permits relatively unrestricted pleasure and a high standard of living. In contrast, restraint implies the tendency to restrict the satisfaction of natural drives with strict social standards.

Previous studies have documented that a society’s cultural values could impact an individual’s psychological processes, such as self-efficacy, judgment, emotion, etc. (Kitayama and Uskul, 2011; Perry, 2012). However, most of them have been limited to comparing a couple of countries (e.g., Di Giunta et al., 2010; Cernas Ortiz, 2022), lacking investigation of more than three. To our best knowledge, Bonneville-Roussy et al. (2019) is the only exception, using data from the PISA 2015 to explore the moderating impacts of two cultural values (individualism and uncertainty avoidance) on the associations between teachers’ teaching practices and student self-efficacy in 16 countries. They found inquiry-based tasks are less effective in predicting the self-efficacy of students from countries that scored highly in uncertainty avoidance, and teacher-led teaching practices are more effective in predicting student self-efficacy if the student are from individualistic countries. Although the relationship between cultural values and student self-efficacy was not directly examined, their findings indicated mixed and complicated associations between multiple cultural values in predicting student self-efficacy.

In addition, there are scant cross-country comparisons in the previous studies that have the benefit of comprehensively considering the influence of cultural values. Six cultural values do not exist in isolation, but rather interact with each other to shape students’ beliefs, attitudes, and behaviors. For example, a student from a culture that values restraint may also value long-term planning, which could potentially increase their self-efficacy. However, if that same student also comes from a culture that values high power distance, they may struggle with expressing their opinions and taking control of their own learning, which could potentially limit their self-efficacy. Therefore, the second purpose of this study was to analyze all of six cultural dimensions using classification and regression tree analysis in order to identify the most influential cultural values, the interactive patterns of cultural values, and the distinct groups of countries at varying levels of student self-efficacy.

### 2.4. Present study

In sum, student self-efficacy is an important factor that significantly contributes to academic achievement and serves as a defining characteristic of successful students. Scholars have made great efforts to make cross-country comparisons of student self-efficacy in order to better understand how to promote it and to decide which nations should be studied in more depth (Wang et al., 2013; Bonneville-Roussy et al., 2019). However, a valid comparison was hard to achieve because it was not easy to satisfy the assumptions of scalar invariance for student self-efficacy measurement tool, particularly in a large-scale assessment. In light of this, the present research makes use of the alignment method in CFA to compare the factor means of student self-efficacy across participating countries and economies in PISA 2018.

Also, as it is still difficult to disentangle the respective contribution of multiple cultural values to student self-efficacy when these values are dynamic and interactive in nature, we use classification and regression tree analysis to segment countries into small groups that share similar Hofstede’s six cultural dimensions, and decompose complicated interactions among these cultural values (Ma, 2005). These are envisaged to answer the following questions:

1. Which countries have the highest (and lowest) student perceptions of self-efficacy?
2. Which cultural values proposed by Hofstede et al. (2005) interactively influence student self-efficacy at the country level?
3. How many distinct groups of countries existed in terms of student self-efficacy?

The significance of our study is multifaceted. First, we employs a novel alignment optimization method to rank the countries and economies considering student self-efficacy which is a research gap has not been addressed. By conducting the analysis, we attempted to offer scientific information based on what the PISA 2018 data says, which could help guide policymakers and education stakeholders toward the right countries or economies to promote student self-efficacy. Second, by using observable cultural dimensions as explanatory variables to identify the distinct groups of countries in terms of student self-efficacy, this study helps educators facilitate student self-efficacy by focusing on those groups of exchange students that may experience cultural shock. Lastly, our study contributes to the broader understanding of the complex relationship between cultural values and student achievement, and may inspire further research in this area to better inform educational policies and practices across diverse cultural contexts.

## 3. Methods

### 3.1. Sample

The current study used information of a sample of 15-year-old students in the PISA 2018 database.^1^ To select a representative sample of students, the PISA team conducted the PISA 2018 survey of schools and students from each country and students in each school in 79 countries using a probabilistic sampling procedure (OECD, 2019a,b).

As participation in the PISA 2018 was optional for countries and schools (OECD), the initial data presented a non-ignorable number of missing values. Thus, we deleted the observations where >20% of the values were missing (Enders, 2003); one country (Norway) was removed, as <20% of every participant’s information was useful; the website did not provide the country of (Kosovo). The filtered dataset contains 521,032 students in 20,827 schools across 77 countries.

### 3.2. Measures of student self-efficacy and cultural values

Student self-efficacy was measured by five items in the PISA 2018 questionnaire. All items share the same question stem: “How strongly do you agree or disagree with the following statements.” Each item has four categories: 1 = Strongly disagree, 2 = Disagree, 3 = Agree, 4 = Strongly Agree, with a higher value indicating a better rate of self-efficacy. Table 1 presents the exact item wording. Cross-cultural values were extracted from the website (https://www.hofstede-insights.com/country-comparison/). The cultural values for our final analytical sample can be found at Table 2. Hofstede’s cultural dimensions data have been widely used in cross-cultural research, and their reliability has been confirmed through multiple studies. Hofstede’s original work in the 1980s was based on a survey of IBM employees in over 50 countries, and subsequent studies have expanded and refined these dimensions using data from different sources (Hofstede et al., 2005). A meta-analysis conducted by Taras et al. (2012) demonstrated that Hofstede’s dimensions have acceptable levels of internal consistency reliability, with Cronbach’s alpha coefficients ranging from 0.65 to 0.75. The validity of Hofstede’s cultural dimensions has also been widely supported in the literature. Numerous studies have demonstrated the predictive and construct validity of the dimensions, showing that they are related to various important outcomes across cultures (Taras et al., 2012). The dimensions have been found to be useful in explaining cross-cultural differences in various domains, including learning (Habók et al., 2021), communication (Puyod and Charoensukmongkol, 2019), and education (Al Hashlamoun, 2021). Given the established reliability and validity of Hofstede’s cultural dimensions, we believe they are appropriate for use in our study to classify countries with differential latent means of student self-efficacy. By employing these dimensions, we are able to account for the complex cultural differences among the countries and economies included in our sample. Furthermore, the dimensions have been used in previous educational research, making them a suitable choice for the present study (see a review Maddux et al., 2021).

**Table 1:** Deleted 34 countries unsatisfied with the GOF indices.

Criterion	# Countries	Country
ω_h_	6	Brunei Darussalam; Iceland; Malta; Mexico; Sweden; Vietnam
TLI	21	Belarus; Brunei Darussalam; Bulgaria; Chile; Costa Rica; Croatia; Czech Republic; Estonia; France; Indonesia; Ireland; Italy; Japan; Malta; New Zealand; Russian Federation; Singapore; Spain; Switzerland; Uruguay; Vietnam
CFI	15	Belarus; Brunei Darussalam; Bulgaria; Czech Republic; France; Indonesia; Ireland; Italy; Japan; New Zealand; Spain; Switzerland; Uruguay; United Arab Emirates; Vietnam
RMSEA	22	Brazil; Bulgaria; Canada; Chile; Costa Rica; Czech Republic; Estonia; Finland; France; Iceland; Indonesia; Ireland; Italy; Japan; Jordan; Luxembourg; Macao; New Zealand; Spain; Tatarstan (RUS); Thailand; Uruguay
SRMR	0	None

Table presents the unique 34 countries that cannot meet the criteria of ωh and GOF indices.

**Table 2:** Non-invariance of student efficacy item intercepts and loadings across 42 countries.

Loadings	Country code
ST188Q01HA	1 2 (3) 4 5 6 7 8 (9) 10 11 (12) 13 14 15 16 17 18 19 20 21 22 23 24 (25) 26 (27) 28 (29) 30 31 (32) 33 34 35 36 37 38 39 40 41 42
ST188Q02HA	1 (2) 3 4 5 6 (7) 8 9 10 11 12 13 14 15 16 17 18 19 20 21 22 (23) 24 25 26 27 28 (29) 30 (31) 32 33 34 35 (36) 37 38 39 40 41 42
ST188Q03HA	1 2 3 4 5 6 7 8 (9) 10 11 12 13 14 15 16 17 (18) (19) 20 21 (22) 23 24 (25) (26) 27 (28) (29) 30 (31) 32 33 34 35 36 37 38 39 40 41 (42)
ST188Q06HA	(1) (2) 3 4 5 6 7 8 9 (10) (11) (12) 13 (14) 15 16 17 (18) 19 20 21 22 23 24 (25) (26) 27 (28) (29) 30 31 32 33 34 35 36 37 (38) 39 40 41 42
ST188Q07HA	1 2 (3) 4 5 (6) 7 8 9 (10) 11 12 13 14 15 16 17 18 19 (20) 21 22 23 24 25 26 (27) 28 29 30 (31) 32 33 34 35 36 37 38 39 40 41 42
Intercepts	
ST188Q01HA	1 (2) 3 4 (5) 6 7 (8) 9 (10) 11 (12) 13 14 (15) 16 17 18 19 20 21 (22) 23 (24) 25 26 27 28 (29) 30 31 32 33 (34) 35 36 37 (38) 39 40 41 42
ST188Q02HA	1 2 3 (4) 5 (6) 7 8 (9) 10 11 (12) 13 14 15 (16) 17 18 19 (20) 21 22 23 24 25 26 (27) 28 29 30 (31) 32 (33) 34 (35) 36 37 (38) 39 40 (41) 42
ST188Q03HA	(1) 2 3 4 5 (6) 7 (8) 9 (10) 11 12 (13) 14 15 (16) (17) 18 (19) 20 21 22 23 24 (25) 26 27 (28) (29) (30) 31 32 33 34 35 36 37 38 39 (40) 41 42
ST188Q06HA	1 2 3 (4) 5 6 7 (8) (9) 10 11 12 (13) 14 15 16 17 18 (19) 20 21 22 23 24 (25) (26) 27 28 (29) 30 31 32 (33) 34 35 36 37 (38) (39) 40 41 42
ST188Q07HA	1 (2) 3 4 5 6 7 (8) (9) 10 11 12 13 14 15 16 (17) 18 19 20 21 22 23 (24) 25 (26) 27 28 29 30 31 32 33 34 35 (36) 37 38 (39) 40 41 42

Countries and economies showing non-invariances are in parentheses. The country code would be found at Table 3.

### 3.3. Analytical procedure

#### 3.3.1. Scale reliability and validity evaluation

Before using the alignment method, it is imperative to evaluate scale reliability and validity. Instead of the popular Cronbach alpha to measure internal consistency, we employed coefficient omega (ω_h_) calculated based on factor loadings and unique variance (Dunn et al., 2014; Flora, 2020). Empirical evidence shows that compared to Cronbach’s alpha coefficient, ω_h_ requires less restrictive assumptions, but provides a more accurate measure of reliability (Zinbarg, 2005; Dunn et al., 2014). A value of ω_h_ greater than 0.7 indicates acceptable reliability (Trizano-Hermosilla and Alvarado, 2016). Moreover, we use confirmatory factor analysis (CFA) to assess the validity. Specially, we tested the unidimensionality of the five-item student self-efficacy scale by conducting CFA for each country/economy robust weighted least squares mean and variance (Bowen and Masa, 2015). Considering the unequal chance of schools being selected within each country, we included the school-level weighting variable (W_FSTUWT_ SCH_SUM in PISA 2018 dataset) in the analysis. A series of goodness-of-fit (GOF) indices are employed to assess mode fit, including the comparative fit index (CFI) and Tucker-Lewis index (TLI) with acceptable value ≥0.90 (Hu and Bentler, 1999), and SRMR and RMSEA with acceptable value ≤0.08 (MacCallum et al., 1996; Hu and Bentler, 1999). The Chi-square statistic was presented, but not utilized to examine model fit because it tends to reject a suitable model with a large sample size (Chen, 2007). The dataset of any country that did not meet the reliability and validity criteria was removed. Whereafter, we evaluated whether the unidimensional model of the student self-efficacy scale met the configural invariance.

#### 3.3.2. Alignment approach for research question one

If the configural invariance was met, we were able to employ the alignment approach to rank countries in terms of student self-efficacy. If the former cannot converge, researchers could switch to the latter. Generally, there are two types of alignment models: FREE and FIXED. FREE alignment method that treats all parameters as free is recommended for more than two-group comparison (Muthén and Asparouhov, 2018). If it cannot converge, scholars could employ FIXED alignment which sets the factor mean of a particular group to zero. Based on the results of the Alignment approach, one could easily tell the items with non-invariance in CFA parameters (i.e., factor loadings and intercepts). Researchers could be statistically confident in making group comparisons if at least 75% of the CFA parameters are estimated to be invariant (Asparouhov and Muthén, 2014).

#### 3.3.3. CART analysis for research questions two and three

CART analysis categorizes associations between the dependent and independent variables based on how those associations emerge across distinct groups (Ma, 2018). To achieve this goal, CART analysis gradually split the pooled sample into homogeneous groups through increasing variations in the values of the dependent variable among groups, which ultimately generates a tree-like map. The first node in the map is called the root node, and the nodes below it are named child nodes. A terminal node is a child node that cannot be further split. The first level of the map represents the strongest relationship between the dependent variable and a specific independent variable. Due to the exploratory benefits of visualizing the impacts of the independent variables on the dependent variables, CART analysis helps to reveal the potential relationships that lack empirical and theoretical support (Ma, 2018).

Our study used CART to partition countries into small homogenous groups that share similar cultural values in terms of factor means of student self-efficacy, and break down complicated interactions among multiple cultural values to pinpoint interaction effects (Ma, 2005, 2018). Data cleaning, CFA, configural measurement invariance examination, and CART analysis were conducted using R version 4.0.3 (R Core Team, 2020), and alignment methods were conducted in M*plus* 8.8 (Muthén and Muthén, 2017).

## 4. Results

### 4.1. Factor structure and configural invariance

Table 1 shows the deleted 34 countries as they fail in at least one of the criteria:ω_h_ ≥ 0.7 (e.g., Vietnam), CFI/TLI ≥ 0.95 (e.g., Italy, Brunei), and RMSEA/SRMR ≤0.08 (e.g., Czech Republic).

The information of the rest of the 42 countries was kept for further analysis as they meet all of the criteria. Table 3 reported the number of schools (11574) and students (308849) and reliability (ω_h_) and validity (CFA) assessments across these countries.

**Table 3:** Descriptive statistics of the sample and measurement quality.

Code	Country	# Schools	# Students	ω_h_			CFA		
					CFI	TLI	RMSEA	SRMR	Chi-Square
1	Albania	326	6,099	0.819	0.989	0.996	0.058	0.017	104.341
2	Argentina	753	17,608	0.750	0.963	0.962	0.078	0.031	390.288
3	Australia	451	10,207	0.778	0.964	0.965	0.063	0.031	516.383
4	Austria	727	11,897	0.776	0.966	0.969	0.071	0.032	259.353
5	Baku (Azerbaijan)	282	6,283	0.898	0.989	0.995	0.079	0.017	149.372
6	Belgium	212	5,819	0.829	0.976	0.989	0.011	0.027	235.402
7	Bosnia and Herzegovina	233	5,636	0.744	0.973	0.984	0.072	0.025	215.448
8	B-S-J-Z (China)	55	6,238	0.807	0.985	0.978	0.066	0.021	262.756
9	Colombia	205	6,436	0.803	0.968	0.974	0.076	0.029	297.170
10	Denmark	215	3,041	0.764	0.958	0.952	0.079	0.036	311.763
11	Dominican Republic	1,088	33,034	0.860	0.977	0.992	0.080	0.026	145.931
12	Georgia	467	12,676	0.822	0.982	0.971	0.076	0.024	131.038
13	Germany	318	4,575	0.734	0.956	0.950	0.079	0.034	165.288
14	Greece	240	6,029	0.756	0.971	0.980	0.078	0.027	182.439
15	Hong Kong	183	5,700	0.815	0.982	0.981	0.076	0.022	166.545
16	Hungary	152	6,288	0.750	0.965	0.967	0.078	0.029	174.325
17	Israel	139	2,991	0.792	0.964	0.965	0.080	0.032	283.546
18	Kazakhstan	183	6,000	0.795	0.969	0.975	0.079	0.036	791.376
19	Korea	611	18,185	0.824	0.977	0.990	0.059	0.029	267.740
20	Latvia	188	6,606	0.812	0.978	0.994	0.078	0.025	147.129
21	Lebanon	210	4,611	0.741	0.967	0.971	0.080	0.029	160.603
22	Malaysia	308	4,553	0.778	0.982	0.971	0.067	0.022	101.482
23	Moldova	308	4,962	0.731	0.963	0.963	0.078	0.030	135.741
24	Montenegro	45	3,763	0.703	0.982	0.980	0.052	0.021	85.667
25	Morocco	269	5,574	0.731	0.968	0.973	0.076	0.028	151.996
26	Moscow Region (RUS)	114	5,157	0.825	0.986	0.990	0.067	0.020	134.759
27	Netherlands	50	3,080	0.774	0.962	0.960	0.070	0.032	196.029
28	Peru	61	6,055	0.778	0.973	0.983	0.068	0.028	63.463
29	Philippines	191	6,010	0.735	0.963	0.963	0.078	0.031	130.006
30	Poland	325	4,326	0.770	0.971	0.979	0.068	0.030	149.456
31	Portugal	187	6,975	0.785	0.994	0.988	0.038	0.013	54.229
32	Qatar	240	5,446	0.785	0.968	0.973	0.069	0.030	220.264
33	Romania	275	5,463	0.755	0.958	0.953	0.069	0.033	241.390
34	Saudi Arabia	187	11,969	0.764	0.980	0.997	0.067	0.025	260.569
35	Serbia	197	4,883	0.752	0.965	0.968	0.069	0.029	179.413
36	Slovak Republic	361	11,981	0.789	0.977	0.990	0.077	0.025	207.383
37	Taiwan	61	1877	0.810	0.980	0.997	0.077	0.024	168.046
38	Thailand	238	5,371	0.826	0.982	0.971	0.077	0.023	164.753
39	Turkey	192	7,134	0.849	0.987	0.980	0.072	0.020	177.199
40	Ukraine	290	8,487	0.729	0.959	0.956	0.069	0.030	218.251
41	United Kingdom	250	5,804	0.775	0.958	0.954	0.071	0.034	625.716
42	United States	187	4,020	0.788	0.961	0.958	0.071	0.033	235.703

Table reports the number of schools (11574) and students (308849) across the 42 countries in which the measurement model had acceptable ωh and GOF indices. United Kingdom contains the regions of United Kingdom (Scotland) and United Kingdom (excluding Scotland).

The descriptive statistics of each item in the student self-efficacy scale were provided in Table 4. On average, students had a positive rating of their self-efficacy.

**Table 4:** Descriptive statistics of student self-efficacy item.

Item	Item wording	*N* (%)	Mean	SD
ST188Q01HA	I usually manage one way or another	283,307 (88.11%)	3.017	0.664
ST188Q02HA	I feel proud that I have accomplished things	282,609 (87.90%)	3.158	0.703
ST188Q03HA	I feel that I can handle many things at a time	282,456 (87.85%)	2.844	0.744
ST188Q06HA	My belief in myself gets me through hard times	282,939 (88.00%)	2.961	0.787
ST188Q07HA	When I’m in a difficult situation, I can usually find my way out	282,967 (88.01%)	3.047	0.693

Table presents the descriptive statistics of the student-efficacy items, including the number of non-missing values, mean, and standard deviation (SD). Item responses were rated on a four-point Likert scale (1 = strongly disagree; 4 = strongly agree).

Consequently, we evaluated the configural invariance of multi-group analyses based on the data from 42 countries. The results of the GOF indices showed that the configural invariance model fits the data well (χ^2^ = 13181.33, *df* = 215, CFI = 0.966, TLI = 0.972, RMSEA = 0.078, SRMR = 0.031).

### 4.2. Alignment method analysis of student self-efficacy scale

We initially used the FREE alignment method to rank the 42 countries and economies being compared. As M*plus* 8.8 did not report any warning about untrustworthy standard errors, there was no need to switch to the FIXED method. Table 2 reports the results of the identified factor loadings and thresholds, together with the respective countries or economies. Countries and economies showing non-invariances are in parentheses.

For example, the loading of item ST188Q01HA is non-invariant in seven countries: Australia (3), B-S-J-Z (China) (9), Dominican Republic (12), Montenegro (25), Moscow Region (RUS) (27), Peru (29), and Portugal (32). That is, the equality of the factor loading condition holds for item ST188Q01HA across 35 other countries. The results of other items’ interceptions and loadings presented at Table 2 can be interpreted similarly. We found that 169 (80.48%) invariant factor loadings of a total of 210 (42*5) parameters. Turning to the intercepts, 156 (74.29%) invariant intercepts were found, which was slightly smaller than the recommended 75% threshold point. Therefore, we were confident in the trustworthiness of the latent mean estimates and comparison for the student self-student scale across countries (Wu et al., 2022).

### 4.3. Student self-efficacy comparison across countries

After testing the reliability and validity of the student self-efficacy scale and the assumptions of the alignment method, we were able to rank 42 countries and economies by comparing the factor mean values of student self-efficacy (Table 5).

**Table 5:** Ranking of the student self-efficacy across 42 countries along with cultural values.

Code	Country	Rank	Factor mean	Power distance	Individualism	Masculinity	Uncertainty avoidance	Long-term orientation	Indulgence vs. restraint
1	Albania	1	1.382	90	20	80	70	61	15
10	Colombia	2	1.077	67	13	64	80	13	83
29	Peru	3	1.021	64	16	42	87	25	46
5	Baku (Azerbaijan)	4	0.999	85	22	50	88	61	22
39	Turkey	5	0.959	66	37	45	85	46	49
25	Montenegro	6	0.958	88	24	48	90	75	20
7	Bosnia and Herzegovina	7	0.907	90	22	48	87	70	44
12	Dominican Republic	8	0.899	65	30	65	45	13	54
36	Serbia	9	0.881	86	25	43	92	52	28
34	Romania	10	0.832	90	30	42	90	52	20
19	Kazakhstan	11	0.826	88	20	50	88	85	22
42	United States	12	0.813	40	91	62	46	26	68
20	Korea	13	0.799	60	18	39	85	100	29
11	Denmark	14	0.791	18	74	16	23	35	70
17	Hungary	15	0.789	46	80	88	82	58	31
35	Saudi Arabia	16	0.752	72	48	43	64	27	14
9	B-S-J-Z (China)	17	0.719	80	20	66	30	87	24
24	Moldova	18	0.704	90	27	39	95	71	19
3	Australia	19	0.702	38	90	61	51	21	71
32	Portugal	20	0.68	63	27	31	99	28	33
41	United Kingdom	37	0.673	35	89	66	35	51	69
37	Taiwan	21	0.667	58	17	45	69	93	49
30	Philippines	22	0.649	94	32	64	44	27	42
15	Greece	23	0.639	60	35	57	100	45	50
31	Poland	24	0.629	68	60	64	93	38	29
18	Israel	25	0.626	13	54	47	81	38	
40	Ukraine	26	0.626	92	25	27	95	86	14
4	Austria	27	0.623	11	55	79	70	60	63
16	Hong Kong	38	0.623	68	25	57	29	61	17
13	Georgia	28	0.598	65	41	55	85	38	32
38	Thailand	29	0.579	64	20	34	64	32	45
6	Belgium	30	0.578	65	75	54	94	82	57
28	Netherlands	31	0.574	38	80	14	53	67	68
33	Qatar	32	0.563	93	25	55	80		
14	Germany	33	0.544	35	67	66	65	83	40
26	Morocco	34	0.515	70	46	53	68	14	25
2	Argentina	35	0.49	49	46	56	86	20	62
21	Latvia	36	0.473	44	70	9	63	69	13
23	Malaysia	39	0.392	100	26	50	36	41	57
8	Slovak Republic	40	0.389	100	52	100	51	77	28
27	Moscow Region (RUS)	41	0.361	93	39	36	95	81	20
22	Lebanon	42	0.235	62	43	48	57	22	10

Table shows the rank of 42 countries and economies in terms of student self-efficacy.

As shown at the table, the rank order of factor means demonstrated that Albania showed the highest factor mean in student self-efficacy, followed by Colombia and Peru. The lowest three countries/economies in student self-efficacy were Slovak Republic, Moscow Region (RUS), and Lebanon.

### 4.4. Description of CART

**Figure 1:**
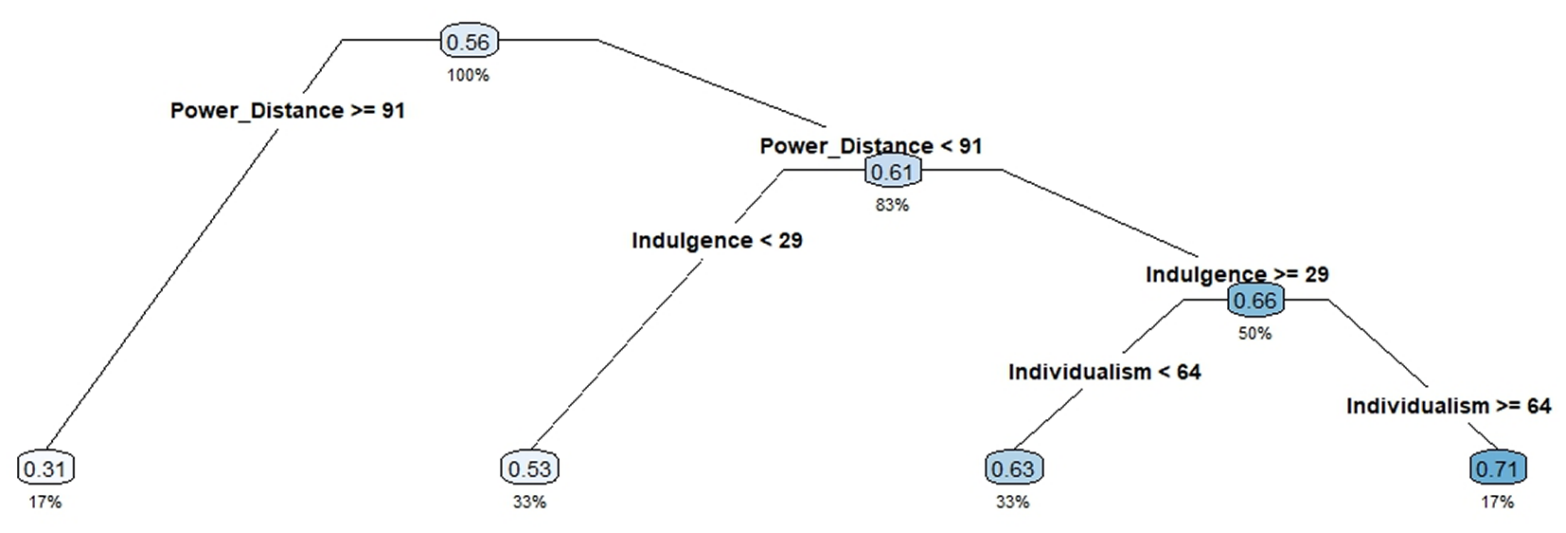
Classification and Regression Trees (CART) of Country-level Student Self-efficacy. Figure presents the CART analysis of student self-efficacy, conditional on cultural values. Value in each node indicates the mean values of country-level student self-efficacy; the value under each node indicates the percent of countries from the original dataset that belong to that node.

Figure 1 shows that CART analysis partitioned countries into nodes with three levels of factor mean scores of student self-efficacy according to the largest reduction in impurity from the root node. No country was eliminated from the data analysis, and the average factor mean score was 0.68. CART analysis was run with six cultural dimensions, but three of them (i.e., uncertainty avoidance, long-term orientation, and indulgence versus restraint) were discarded.

The root node was first split according to the power-distance dimension (this dimension had the largest reduction in impurity from the root to its child nodes). The left child node became a terminal node (G1); it contained seven countries (17%) that scored at least 91 and whose average factor mean score was 0.31. The right child node contained 35 countries (83%) whose power-distance scores were less than 91 and whose average factor mean score was 0.61. Among those countries, 14 (33%) with indulgence scores less than 29 became a terminal node with an average factor mean score of 0.53 (G2). The other 21 countries (50%), which had an average factor mean score of 0.66, were split further according to the individualism dimension. Fourteen countries (33%) with individualism scores less than 64 had an average factor mean score of 0.63 (G3), and seven (17%) with individualism scores equal or greater than 64 showed an average factor mean score at 0.71 (G4).

### 4.5. Description of cultural dimensions in terminal group

Table 6 reports factor mean scores in three significant cultural dimensions (power distance, individualism versus collectivism, and indulgence versus restraint) in each of the terminal nodes from low (G1) to high (G4). The root node (G0) is also present for the purpose of comparing a particular terminal node with the average value of factor mean scores.

**Table 6:** Means of factor mean student self-efficacy in terminal nodes.

Cultural values	G0	G1	G2	G3	G4
Power-distance	68.05	94.01	78.07	61.31	35.71
Individualism	40.82	32.14	31.43	33.69	81.57
Indulgence	37.53	28.83	19.50	48.69	59.57

G0 is the root node, G1-4 are the terminal nodes in Figure 1.

Descriptive statistics showed that, for the terminal node with the lower factor mean scores (G1), average power-distance scores were extremely high (94.01) and both the individualism (32.14) and indulgence (28.83) scores were the second-to-last across all groups. Conversely, countries in the terminal node with the highest average value of factor mean scores (G4) were dominated by a low power distance (35.71), extremely individualist (81.57), and moderately indulgent culture (59.57). The countries in terminal nodes G2 and G3 shared similar high power distance (78.07 and 61.31, respectively) and collectivistic (31.43 and 33.69, respectively) cultures, but showed very different attitudes regarding an indulgent lifestyle (19.50 vs. 48.69).

## 5. Discussion

By applying the alignment method in CFA to a large-scale international assessment (i.e., PISA 2018), this study answered our research questions regarding the comparisons of student self-efficacy across 42 countries and economies and the impact of cultural values on shaping student self-efficacy. We found that: (1) based on their factor mean scores, Albania, Colombia, and Peru comprised among the top league of the student self-efficacy assessment and Slovak Republic, Moscow Region (RUS), and Lebanon comprised the bottom; (2) Using Hofstede’s six cultural dimensions as predictors, CART analysis identified that three of the six cultural dimensions engaged one another in a complex interactive manner to impact student self-efficacy in a way that is far more complicated than that which many traditional statistical methods such as ANOVA can usually recover (Figure 1). Moreover, power distance was the most critical cultural dimension shaping student self-efficacy. (3) CART analysis also characterized all 42 countries into four groups that were represented as four homogenous terminal nodes (Figure 1 and Table 6).

### 5.1. Ranking of country-level student self-efficacy

The results of this study supported a one-factor structure of the student self-efficacy measurement tool, as well as satisfactory reliability indices (ω_h_ ≥ 0.70) across most of the countries and economies that participated in PISA 2018, which is consistent with previous research (Pepper et al., 2018; OECD, 2019a,b). A new addition of this study is the ranking of countries and economies by the factor means of student self-efficacy. This contribution recognized Albania as a country whose students had the highest sense of self-efficacy. What did students in Albania do differently? According to the findings of OECD (2019a,b), from 2009 and 2018, student reports disciplinary climate generally improved (López et al., 2022; Zhu and Teng, 2022) and were most likely to co-operation amongst their peers rather than competition (Rudolf and Lee, 2023), and teachers were more passionate in their teaching and continued teaching until students understand (Ortan et al., 2021; Liu and Wang, 2022). The results of the current research also provide a framework for ranking countries according to country-level student self-efficacy. This new addition to the body of knowledge has consequences for educators and policymakers in terms of the countries to look to for promoting better student self-efficacy(Al-Abyadh and Abdel Azeem, 2022; Yang et al., 2022).

### 5.2. Cultural values shaping student self-efficacy

#### 5.2.1. Understanding the power-distance dimension

Our findings suggest that compared with other cultural dimensions, power distance is the most important predictor of student self-efficacy across varying levels, suggesting that it has a pervasive influence across different levels of student self-efficacy. Findings showed that countries with extremely high power distance (G1) have below-average scores in student self-efficacy, which is documented by previous studies. One international study found that Filipino students usually experience higher anxiety and lower math self-efficacy than American and Korean counterparts (Ahn et al., 2016). This could be explained by the large power distance in Filipino society that strictly requires students to follow classroom rules and then generates great social stress. To improve self-efficacy, it is important to address power distance and create more egalitarian and inclusive educational environments.

#### 5.2.2. Understanding the indulgence dimension

Distinct patterns were detected after the most substantial partition at the first level of prediction. The indulgence dimension behaves uniquely, appearing only for countries reporting a lower score on the power-distance dimension. In the literature, indulgent culture has mixed effects on student self-efficacy. On one side, an indulgent culture can negatively impact student self-efficacy through low expectations and a lack of challenge (Reyes, 2019). Students need opportunities to push themselves beyond their comfort zones to develop a sense of mastery and confidence in their abilities (Zhang et al., 2018). However, in an indulgent culture, students may not receive the feedback and support necessary to challenge themselves and develop their skills. Without the opportunity to experience success through effort and perseverance, students may struggle to develop a strong sense of self-efficacy (Cernas Ortiz, 2022). However, on the other side, students who come from indulgent societies are more likely to believe that they can achieve their goals and pursue their passions, which can increase their sense of self-efficacy. Additionally, indulgent societies tend to be more tolerant of failure and encourage risk-taking (Alipour and Yaprak, 2022). When students are not afraid to make mistakes and take on challenges, they are more likely to develop a sense of competence and confidence in their abilities.

Our study supported the second case. This discrepancy might be explained by the interaction between power distance and indulgence. Compared to G3-4, G2 scored higher in the power-distance dimension (Table 6), which could increase student anxiety and depression caused by the large power distance. However, these negative perceptions could be mitigated by a relaxed lifestyle, such as creating more opportunities to enjoy life and have fun (Smith et al., 2007; Park et al., 2022). Indeed, the gratification of positive desires is fundamental to Hofstede’s interpretation of Indulgence in cultural dimension theory (Hofstede et al., 2005, p. 281).

#### 5.2.3. Understanding the individualism dimension

Lastly, at the third level of the regression tree, the individualism dimension was very relevant for the groups of countries with moderate (G3) and high (G4) levels of student self-efficacy. Specifically, students in countries that scored <64 in the individualism dimension (G3) held weaker beliefs in their ability to fulfill their academic tasks than their counterparts in countries with scores of ≥64 (G4). Two reasons may explain this phenomenon: (1) the high self-efficacy scores may reflect cultural demands for personal responsibility. In individualistic societies, students are often expected to take responsibility for their own lives and academic success (Wang et al., 2020; Tan et al., 2021). This can foster a sense of control and agency, as students feel that they have the power to shape their own future; (2) compared to students from collectivistic societies, those from individualistic societies are less negatively susceptible to failure experiences (Ahn et al., 2016). Taking risks and pursuing new ideas are often seen as positive traits for an individualistic culture, as they can lead to innovation and progress (Tran, 2019; Chang, 2021). This can facilitate a culture of experimentation and creativity, which can increase self-efficacy by mitigating students’ fear of failure.

Moreover, G4 also scored very low on the power-distance dimension (35.71; Table 6), reflecting the critical fact that cultural values do not exist in isolation, but rather interact with each other to shape student self-efficacy (Yang et al., 2020). Students from individualistic cultures and lower power distance were more likely to have higher levels of self-efficacy beliefs, and the interaction of these two cultural values could lead to a stronger sense of self-efficacy. These findings may be due to the greater emphasis on personal achievement and autonomy in individualistic cultures, as well as the reduced reliance on authority figures in cultures with low power distance.

#### 5.2.4. Summary

Although the rest three cultural dimensions of femininity, uncertainty avoidance, and long-term orientation have been potentially related to student self-efficacy (e.g., Bonneville-Roussy et al., 2019), the CART analysis discarded them in our study. However, we cannot state these cultural dimensions are not relevant. Indeed, because our final analysis sample size is small (42 countries), the dropped dimensions may be critical in unincluded countries.

In sum, the current research recovered a discernible pattern in the way three cultural dimensions are able to significantly shape student self-efficacy. The CART analysis underlines that these dimensions are not conceptually well orderly, linear, or closely related; rather, they offered distinct contributions to predict student self-efficacy. These findings have important implications for educators and policymakers, as they suggest that cultural factors can have a significant impact on student motivation and achievement. Educators should be aware of these cultural differences and strive to create learning environments that are inclusive and empowering for all students, regardless of their cultural backgrounds (Day and Beard, 2019; O’Leary et al., 2020).

### 5.3. Limitations and further research

This study is subject to several limitations, which also provide avenues for further research. First, Albania ranks first among the participating countries and economies in PISA 2018. However, we did not provide empirical evidence that would justify Albania being placed in that position, which requires further field research. Investigating the specific factors that contribute to Albania’s high ranking in student self-efficacy would help provide a more nuanced understanding of the underlying causes and potentially inform policy recommendations for other countries.

Second, a large sample size is crucial for CART analysis, but our study had a relatively small sample, which might affect the findings’ robustness. Subsequent research should consider incorporating more countries and economies to expand the sample size and improve result generalizability. Furthermore, conducting replication studies using alternative datasets like the Trends in International Mathematics and Science Study (TIMSS) or the Progress in International Reading Literacy Study (PIRLS) would support the validation and verification of our findings.

Countries with low student self-efficacy may also benefit from more studies into the function of educational policies and initiatives that foster self-efficacy. This would aid in providing actionable suggestions on how to create a learning environment that is supportive of students’ self-efficacy in multiple cultural settings. Lastly, extending the study’s scope to include additional psychological categories like motivation, resilience, or well-being might help create a more complete picture of the link between cultural values and other elements of student achievement.

### 5.4. Implications

The findings in the current study highlighted the importance of cultural values in shaping student self-efficacy across countries. Thus, the present study carries several theoretical and practical implications. Theoretically, our findings contribute to the literature by demonstrating that not all cultural values are related to student self-efficacy. The identification of three cultural values (i.e., power distance, indulgence, and individualism) allows for a broader understanding of the complicated process and mechanisms that buffer the impact of cultural values on student self-efficacy. These findings also demonstrated that research on self-efficacy should take cultural differences into account (Gebauer et al., 2021). Cross-country comparisons are not often feasible, but the function of cultural values should also be considered inside individual countries (Bonneville-Roussy et al., 2019). Consequently, for countries that are more ethnically heterogeneous (e.g., in North America), it is necessary to have a deeper theoretical grasp of the nature of self-efficacy in relation to different cultural groups (Khine and Nielsen, 2022).

Practically, this study employed the alignment method to rank 42 countries and economies using the PISA 2018 dataset, offering valuable insights for policymakers and education stakeholders. By identifying countries or economies with lower student self-efficacy levels, our findings can inform targeted interventions and policies aimed at enhancing self-efficacy within these regions. This approach allows for a more focused allocation of resources and the development of tailored strategies that address the unique cultural factors influencing self-efficacy in each country. In addition, the ranking can facilitate international collaboration and knowledge sharing between countries with varying self-efficacy levels, leading to the development of best practices and innovative solutions to improve student self-efficacy across diverse cultural contexts. This study also informs educators in secondary education institutes on the internationalization of academic exchanges such that host institutions can better support exchange students’ academic success (Khine and Nielsen, 2022). We used Hofstede’s six cultural dimensions as explanatory variables to predict country-level student self-efficacy. These cultural dimensions are observable and identifiable, so teachers and principals could easily focus on the exchange student groups that may experience cultural shock. For example, for exchange students from a culture with high power distance, they may struggle to adapt to a culture with low power distance where students are expected to take a more active role in their learning. To build their self-efficacy, the student may need to seek out opportunities to speak up and participate in class discussions or group projects. Similarly, for students from a culture that places a high value on collectivism, they may struggle to adjust to a culture that emphasizes individualism. In this case, the student may need to build their self-efficacy by learning how to advocate for themselves and assert their individual needs and preferences. Overall, understanding the cultural dimensions may help exchange students navigate their new cultural context and develop the skills and confidence they need to succeed in their studies.

Finally, as teacher-student interaction is the basic social relationship in schools, our study underlined the need to increase teachers’ awareness of the roles that cultural values play in student cognitive behaviors (Bonneville-Roussy et al., 2019). Schools and governments would be the best agents to launch initiatives aiming to boost a basic understanding of how teachers’ words and actions could impact exchange students’ self-efficacy in terms of their diverse cultural backgrounds (Margolis and Mccabe, 2006). This would be particularly useful for students with low self-efficacy from stronger power distance and less indulgent countries (G1-2).

## Data availability statement

Publicly available datasets were analyzed in this study. This data can be found at: https://www.oecd.org/pisa/data/2018database/.

## Author contributions

RJ developed the ideas. RJ, RW, YX, and MZ jointly wrote the manuscript. All authors contributed to the article and approved the submitted version.

## Funding

This article was supported by the National Social Science Fund of China under the project “A longitudinal study of teacher identity and professional development of non-teacher-oriented graduates from high-level comprehensive universities” (Project No. BHA210136).

## Conflict of interest

The authors declare that the research was conducted in the absence of any commercial or financial relationships that could be construed as a potential conflict of interest.

## Publisher’s note

All claims expressed in this article are solely those of the authors and do not necessarily represent those of their affiliated organizations, or those of the publisher, the editors and the reviewers. Any product that may be evaluated in this article, or claim that may be made by its manufacturer, is not guaranteed or endorsed by the publisher.

## Footnotes

^1^https://www.oecd.org/pisa/data/2018database/
